# Neural bases of freedom and responsibility

**DOI:** 10.3389/fncir.2023.1191996

**Published:** 2023-06-02

**Authors:** Agnès Gruart, José M. Delgado-García

**Affiliations:** Division of Neurosciences, Universidad Pablo de Olavide, Seville, Spain

**Keywords:** consciousness, free will, internal world, prefrontal cortex, unconscious processing, behavior

## Abstract

This review presents a broad perspective of the Neuroscience of our days with special attention to how the brain generates our behaviors, emotions, and mental states. It describes in detail how unconscious and conscious processing of sensorimotor and mental information takes place in our brains. Likewise, classic and recent experiments illustrating the neuroscientific foundations regarding the behavioral and cognitive abilities of animals and, in particular, of human beings are described. Special attention is applied to the description of the different neural regulatory systems dealing with behavioral, cognitive, and emotional functions. Finally, the brain process for decision-making, and its relationship with individual free will and responsibility, are also described.

## Introduction

Current Neuroscience studies how the brain generates and regulates behavior, emotional states, and mental activity in animals and humans. But this has not always been so. Until the 1950s, the study of the brain focused on the cellular organization of nervous tissue, on the functional aspects of action potentials, and on all the experience gained from clinical studies related to the various pathologies that affect the brain. In the successive years, the appearance and refinement of many different molecular, cellular, and physiological techniques made possible the experimental approach to functional aspects such as learning, memory, attention, feelings, mental activities, and social interactions. Thus, while in the second edition (1985) of the Principles of Neural Science, by Kandel et al. (2013) (a reference work in the field of Neuroscience), it is indicated that “the neural science is required for understanding human behavior, because all behavior is an expression of neural activity,” in the fifth edition of the same handbook (2013) the objective of neuroscientific studies changes considerably: “the central focus of biology has shifted to neural science and specifically to the biology of mind.” In fact, today there are excellent reports and conceptual proposals dedicated to the study of conscious and unconscious mental processes, as well as the emotional organization of our “internal world” (Taylor and Villa, 2001; Lisman, 2015; Tononi et al., 2016; Buzsáki and Llinás, 2017; Dehaene et al., 2017). The latter concept refers to the subjective sensation that our thoughts, desires, emotions, and feelings represent. The internal world is not exclusive to human beings, not even to primates, but rather it develops in definition and complexity along the evolutionary scale of vertebrates, mainly mammals. At the same time, aspects as diverse as the neural foundations of free will and decision-making, or those that make social interactions possible, such as language and economic exchanges, have also come under the study focus of neuroscientists (see Glimcher, 2004). There are also many reports considering in detail some important aspects of brain functions in relation to philosophical, cultural, and legal concepts such as the freedom and predetermination of our acts, or the contribution of free will in the decisions we make (Searle, 1997; Shadlen and Roskies, 2012; Lavazza, 2016), so such aspects will not be repeated here. In contrast, we will focus on the description of the main contributions of Neuroscience to a better knowledge of our manner of behaving, thinking, and feeling in a way understandable for non-neuroscientists participating in this Research Topic.

## The brain as a generator and controller of all types of behaviors

One function of the brain is to produce behaviors—that is, to activate the muscles of the organism to act on the surrounding space, to look at and to adapt to new spatiotemporal niches, or to interact with our fellow human beings. For Sherrington, all we can do is to activate muscles, whether to cut down a forest or to recite a poem. From a purely motor point of view, behavior can be explained quantitatively by applying the laws of biomechanics, all based on Newton’s second law of motion: the driving force (*F*) needed to move an object depends on its mass (*m*) and on the acceleration (*a*) that you want to impose on it (*F* = *ma*). Naturally, when in a behavior you have to activate many muscles in a certain order and with displacements and forces adjusted to what you want to achieve (for example, holding in your hand a glass of water to drink without letting it fall to the ground) you need to generate a complex program of actions well-organized and carried out in an organized manner. Specific areas of the brain deal with this, ranging from the sensory cerebral cortices, where perceptive and predictive aspects are identified, to the premotor and motor cortices where movement is programmed and the necessary orders are given. The premotor and motor cortices are located in the caudal portion of the frontal lobe, where the necessary motor orders are designed for the correct performance of the most complex movements, particularly those of the hands, face, and larynx. All these motor orders have to reach the motoneurons (the final common pathway, in the words of Sherrington) where all the neural information is integrated and from where it is transmitted to the muscles so that they move the hands to grasp a glass, the legs to walk, or the oral cavity to speak. Motoneurons are highly specialized neurons whose somas (the neuronal body) are located in the brainstem or spinal cord and whose axons (a prolongation originating from the neuronal soma) leave the nervous system and, through motor nerves, arrive, in a selective and organized way, to all the muscles of the organism. Thus, neural motor commands are encoded by the brain in the frequency and timing of action potentials generated at neural motor centers. These neural codes are transformed in the muscles into the force necessary to carry out the aimed motor acts (Delgado-García, 2000; Kernell, 2006).

An important aspect is that behaviors are public-facing. Most behaviors are seen and understood by their observers. For example, we can see and determine whether a dog is walking, running, or lying down. Thus, we can interpret what it does. But also through the behaviors that we observe we can interpret (read) the mind of the observed. If the dog shows us its teeth, we can interpret that it is threatening us and that it might attack. In this interpretative function of the intentions (mind) of the dog or of a person who threatens us, not only the pathways and visual centers are involved, but also neuronal structures involved in more-complex visual functions, located mainly in the superior temporal sulcus, which is intimately connected with various areas of the prefrontal cortex (mainly the anterior cingulate, medial prefrontal, and supraorbital prefrontal cortices). In this way, our behaviors involve motor orders so that the movements can be carried out in accordance with the laws of biomechanics, but these will in turn be colored by our motives and intentions: for example, walking to go to a restaurant or to a theater. The reason for doing the first activity is hunger, which is triggered by, among other possibilities, a drop in glucose concentration detected in the hypothalamus. In contrast, the motivation for the second action is our interest in a given new play or possibly our long affection for Shakespeare’s dramas.

We should also remember that carrying out a behavior is not the same as thinking about it. In fact, a possible evolutionary origin of mental activity is the “invention” of behaviors that are planned internally but are not carried out (Llinás, 2001). Imagine a lynx that tries to catch a rabbit because it is hungry, but fails. The lynx can review in his internal world, over and over again, the hunting strategy and how to improve it for the next time. If in the active carrying out of movements there is the intervention of brain centers where these are planned, and motor centers which generate the motor orders that are transmitted to the motoneurons and from these to the muscles, in thought movements only the centers that plan the movement are activated (mainly, the sensory areas in which tactile sensations, images, and sounds are imagined, as well as the premotor and parietal cortex areas in which the motor acts to be performed are planned).

## The brain as a generator and controller of mental activities

Thanks to the studies of pioneers such as Kleitman, Dement, Hobson, Jouvet, and many other researchers, we know in detail the phases of wakefulness and sleep that occur throughout the circadian rhythm, also called nictemeral cycle (Dement and Kleitman, 1957; Jouvet, 1980; Hobson, 1988; Kandel et al., 2013; Cobb, 2020). Throughout a complete day we go through a phase of wakefulness and alertness, a phase of light sleep, and a phase of deep sleep. The waking phase is characterized by an electrical activity of the cerebral cortex of a variable frequency (1–120 Hz) and that is peculiar to each cortical zone. The light sleep phase is normally divided into four periods characterized by the presence of slow waves and the so-called sleep spindles. Finally, the deep sleep phase is also called paradoxical sleep because while cortical activity is similar to that which occurs during wakefulness, and there are also rapid eye movements, the individual is completely asleep, with a considerable muscle relaxation. While nightmares without visual content usually occur during light sleep, dreams with strong visual content usually appear during the paradoxical sleep phase. For this reason, in some theories it has been assumed that the waking state is an evolutionary phenomenon that is generated from the paradoxical dream, but with the difference that during wakefulness we can distinguish reality from dreams, except in pathological situations such as during hallucinations or delusions.

Within the awake or waking state, there are differences depending on the level of attention and whether it is focused on sensory (perceptive) aspects, on the elaboration of behaviors (motor designs), or on cognitive processes (mental activity). A good example is the long process involved in learning to drive. The first days one is very attentive to all kinds of sensory stimuli (visual, tactile, auditory), with high muscle tone and a certain imprecision and abruptness in movements; in short, with a high degree of attention to everything that happens. But, over time, this level of attention relaxes and it is possible to attend to other tasks not directly related to driving, and motor activity becomes less tense and, above all, it flows unconsciously.

**FIGURE 1:**
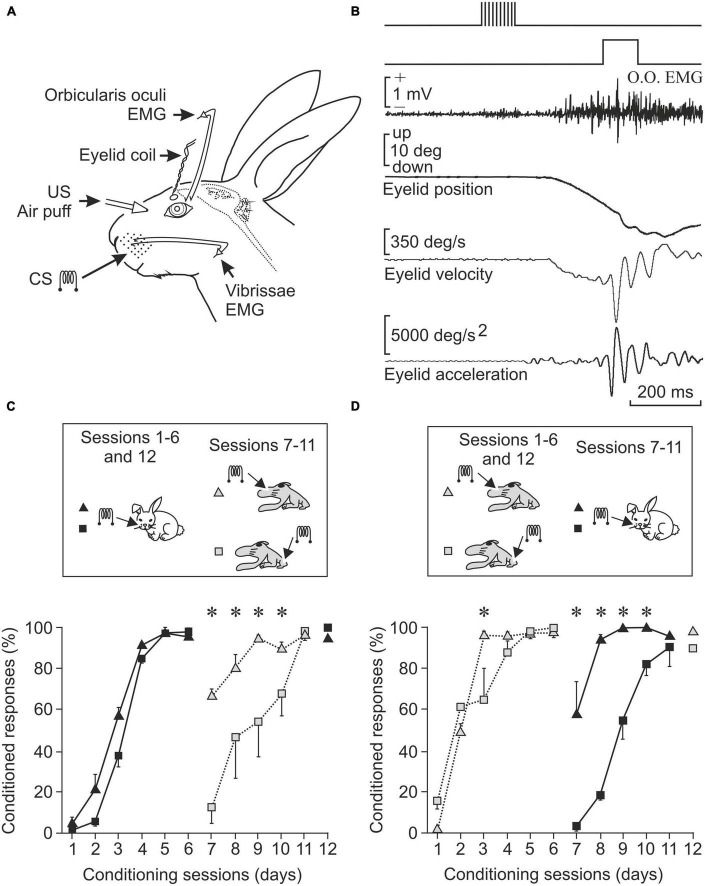
The primary sensory areas introduce into the brain maps and information of interesting aspects present in the outside world. **(A)** Experimental design to record the electrical activity of the orbicularis oculi muscle (responsible for closing the lids) and the vibrissae musculature, as well as the position of the upper eyelid of the experimental animal. Electrodes implanted to stimulate the vibrissae at 200 Hz (CS, conditioned stimulus) and the cornea with a puff of air (US, unconditioned stimulus) are also shown. **(B)** Shown from top to bottom are CS, US, orbicularis oculi muscle activity, and eyelid position, velocity, and acceleration. **(C)** Conditioned responses in animals stimulated first in the vibrissae and then in areas of the primary somatosensory cortex corresponding to the vibrissae or to the hind paw. Note that learning is best maintained if the somatosensory cortex area corresponding to the vibrissae is stimulated. **(D)** Opposite experiment. Conditioned responses in animals stimulated in the somatosensory cortex area of the vibrissae or in that of the hind paw and then directly in the vibrissae. The acquired conditioning is better preserved when the preceding stimulus was in the vibrissae cortex (*P < 0.05). Taken with permission and modified from Leal-Campanario et al. (2006) (Copyright 2006 National Academy of Sciences).

The phenomena indicated above are generated by specific areas of the central nervous system. For example, the so-called primary cerebral cortices receive specific information from each sensory modality: visual, tactile, auditory, etc. Today it is known that if we stimulate the visual cortex with an appropriate program, visual sensations can be induced which are assumed to occur outside of us, not in our brain. In this sense, and in an experiment carried out by our group, we demonstrated that stimulation of the vibrissae of an awake rabbit at 200 Hz can be replaced by the same stimulation applied to the corresponding area of these vibrissae in the somatosensory cortex of the animal (Leal-Campanario et al., 2006). That is, in both cases, the individual perceives the sensation as located outside. We know this from the person stimulated by his statements, or from the experimental animal through learning tests in which stimulation is applied to the vibrissae or to the somatosensory cortex, indistinctly (Figure 1).

Since the end of the 19th century it has been known that electrical stimulation of the premotor and motor cortex evokes facial or limb movements, depending on the area stimulated; even the stimulation of specific areas of the striatum can induce more-complex movements such as turns of the whole body or taking a few steps in an organized way. In patients being intervened for brain surgery, memories and emotional situations can be induced by activating areas of the temporal lobe, while in laboratory animals the emotional state can be altered by activating the amygdaloid nucleus (Delgado García et al., 1998; Kandel et al., 2013; Cobb, 2020). The amygdaloid complex is a set of nuclei located inside the rostral temporal pole and related to functions such as attack/flight responses and aversive-type stimuli and learning. At a higher level, the course of cognitive processes can be changed, for example, during the performance of a learning task, through the electrical activation of specific areas of the prefrontal cortex; or likewise, the degree of attention to a task can be increased if positive reinforcement areas, such as the accumbens nucleus or the medial septum, are activated (see below). The accumbens is a subcortical nucleus located in the ventral striatum that is related to the reward and gratification mechanisms of appetitive-type behaviors. On the other hand, the septal nuclei are closely related to the olfactory pathway. They have several subdivisions. In particular, reference is made here to the medial septum, closely related to the hippocampus, onto which it projects (Olds, 1958; Wise, 2002; Jurado-Parras et al., 2012; Vega-Flores et al., 2014).

Information collected across the years (Delgado García et al., 1998; Llinás, 2001; Kandel et al., 2013; Cobb, 2020) indicates that anesthesia, experimental lesions, pharmacological manipulations, or clinical pathologies of the areas indicated above produce effects contrary to their stimulation. Thus, bilateral anesthesia of the visual nerve pathways or of both lateral geniculate nuclei of the thalamus results in loss of vision, and one can say that one cannot see although all other functions are preserved. Anesthesia of the medial geniculate nucleus causes hearing loss, and so on with other sensory centers. In relation to the motor centers, anesthesia of the motor cortex produces important deficits in the generation of movements, mainly facial and manipulative ones. But a different level of effects is produced if we inactivate centers of a cognitive nature. For example, anesthesia of the reticular nuclei of the thalamus produces a total disconnection of the waking state and the induction of a neurological coma, a sign that this structure is essential in the generation of the conscious state (Llinás, 2001). Also, the inactivation of structures such as the amygdaloid nuclei and specific areas of the prefrontal cortex alters the social behavior of both laboratory animals and people (Gangopadhyay et al., 2020). The prefrontal cortex occupies the most rostral portion of the frontal lobe, located anterior to the motor and premotor cortices. It is usually subdivided into medial, supraorbital, and lateral, although there are other types of subdivision. It is related to the elaboration of complex motor and cognitive functions such as decision-making and other executive functions.

## Brain mechanisms underlying the acquisition of new motor and cognitive abilities

The classical conditioning of the corneal reflex is an interesting experimental model that involves practically all the aforementioned structures, with each of them contributing according to its peculiar functional characteristics (Gormezano et al., 1983; Thompson, 1988; Christian and Thompson, 2003). Normally, a puff of air applied to the cornea induces a reflex closure of the eyelids; that is what the corneal reflex consists of. In contrast, the presentation of a sound that is not very intense produces no response from the eyelids. However, if we present the same sound several times followed by a puff of air in less than a second, we will end up with the appearance of a palpebral movement (called a conditioned response) in response to the sound. This type of classical or Pavlovian associative learning is widely used in the study of learning mechanisms because its functional properties are very similar in all species studied, from rodents to primates, including humans. Our research group has studied the neural bases of this experimental model for the last 30 years [see details and references in Parras et al. (2022)]. On the one hand, the sensory aspect involves the participation of the somatosensory and auditory cortices, because these are the cortical areas that detect the puff of air (following the activation of corneal mechanoreceptors) and the sound. From the motor point of view, the palpebral response is elaborated mainly in the facial motoneurons, but these motoneurons receive the corresponding motor orders from the red nucleus (Pacheco-Calderón et al., 2012; Basile et al., 2021), located in the midbrain, and from the motor cortex (Ammann et al., 2016). The refinement or improvement of the performance of the acquired learned movement occurs probably in the cerebellum (Welsh and Harvey, 1989).

**FIGURE 2:**
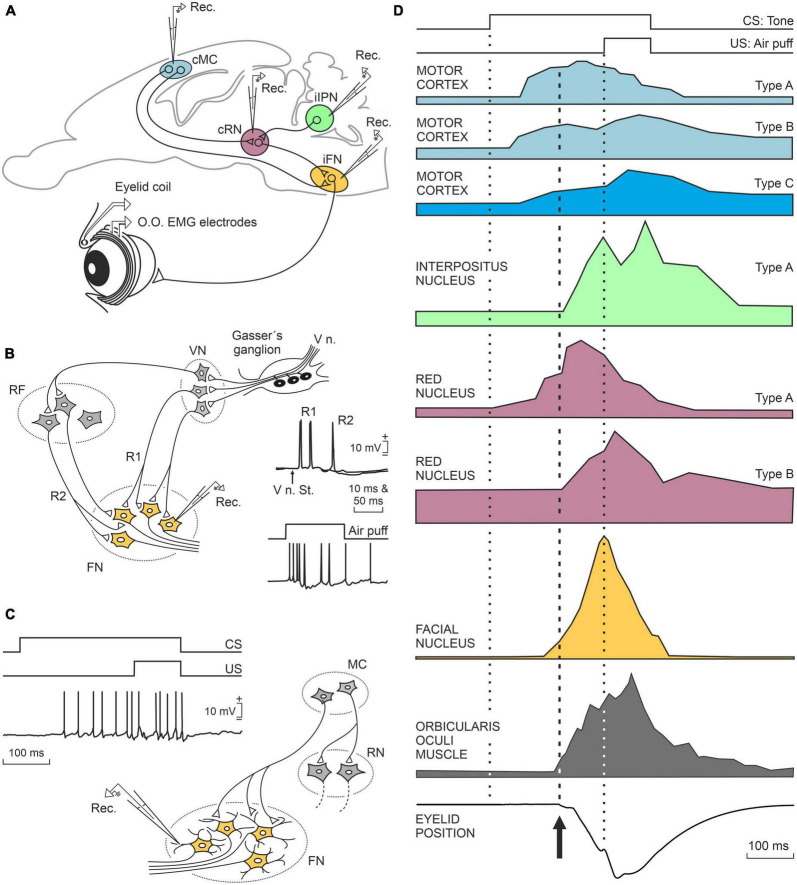
A representation of the neuronal activity of the main motor centers that participate in the generation of conditioned eyelid responses. **(A)** Location of the motor centers illustrated in **(D)**. **(B)** Schematic diagram of the bisynaptic corneal reflex and how the afferent neural pathways to the facial nucleus terminate in the somata of facial motor neurons. **(C)** Diagram illustrating that in the case of conditioned responses, the afferent pathways terminate in the distal dendrites producing weaker responses than those evoked in the corneal reflex. **(D)** From top to bottom are illustrated the experimental design with the presentation of the conditioned stimulus (CS, tone) and the unconditioned stimulus (US, airpuff presented to the cornea) and the neuronal responses recorded in the motor cortex, interpositus nucleus of the cerebellum, red nucleus, facial motoneurons, orbicularis oculi muscle, and conditioned (learned) closure of the eyelids. Taken with permission from Parras et al. (2022).

**FIGURE 3:**
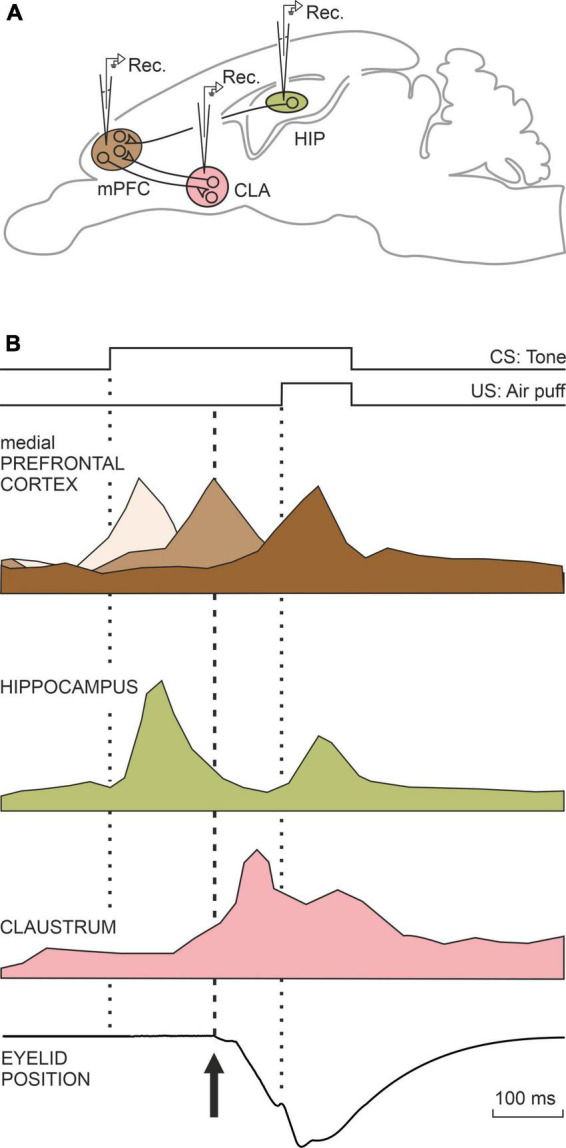
A representation of the neural activity of three neural centers that do not participate in the generation of conditioned motor responses, but rather in the cognitive aspects related to this type of learning **(A,B)**. The medial prefrontal cortex is involved in determining the interval between the onset of CS and the onset of the US. The hippocampus determines the salience or relevance acquired by the CS among many other stimuli present during the conditioning test. The claustrum also appears to be involved in cognitive aspects unrelated to conditioned responses. Taken with permission from Parras et al. (2022).

Many other brain centers participate in the acquisition and memory of this apparently simple movement, but in this case, they are mostly involved in non-motor, cognitive aspects of the acquisition and storage processes, such as the hippocampus, the prefrontal cortex, and the claustrum. In first place, the activity of hippocampal neurons seem not to be related to the acquired motor processes, but cognitive ones such as the salience or relevance of the conditioned stimulus—i.e., the tone (McEchron and Disterhoft, 1997; Delgado-García and Gruart, 2006). Hippocampal activity is also related to the memorization of this type of associative learning (Neves et al., 2008). Also in relation to classical eyeblink conditioning, the prefrontal cortex is concerned with short-term memory, determining the interval between tone and airpuff, as well as inhibiting any other movement during the generation of the learned response (Siegel et al., 2012; Caro-Martín et al., 2015). Finally, the claustrum has also been proposed as being involved in non-motor aspects of learning phenomena (Citri and Barretta, 2016). The claustrum is a peculiar lamellar neuronal structure that extends beneath the lateral cerebral cortex and whose neurons are interconnected with most sensory, motor, and cognitive cortical regions. Crick and Koch (2005) had already pointed out that the nucleus of the claustrum could be the integrating seat of the cognitive functions (of the soul, in his words). Whether this is the case or not, the activation of the claustrum during this learning occurs after the learned or conditioned response has been produced, with a notable increase in electroencephalographic synchronization with the cerebral cortex, mainly the prefrontal (Reus-García et al., 2021). A summary of the joint activity of all these motor and cognitive centers is shown in Figures 2, 3.

## Unconscious and conscious processing carried out by neural circuits

As early as 1860, Hermann von Helmholtz proposed, based on his studies of the speed of propagation of the nerve impulse, that in the brain there was unconscious processing (“unconscious inference”) of sensory information until it reached the conscious level or elaborated a complex response. Already by the beginning of the 20th century, Sigmund Freud promoted the concept of unconscious processing of certain affections and/or memories. From a physiological point of view, Cannon (1932) noted how most metabolic processes (from respiratory rate to blood glucose levels) are regulated automatically, through feedback mechanisms, leaving the conscious attentional processes free for interactions with our physical and social environments. Three different types of unconscious processing of neural information can be characterized (Kandel et al., 2013): the implicit unconscious refers to the undeclared, manipulative and non-verbal memory that is elaborated mainly in the cerebellum and large areas of the striatum; the dynamic unconscious related to emotional conflicts, repressed thoughts, and sexual or aggressive desires subtracted from consciousness, elaborated in undefined areas of the limbic system; and, finally, the preconscious unconscious refers to the preparation of executive plans (complex behaviors, decision-making), attributed to the parietal, prefrontal, and premotor cortices.

**FIGURE 4:**
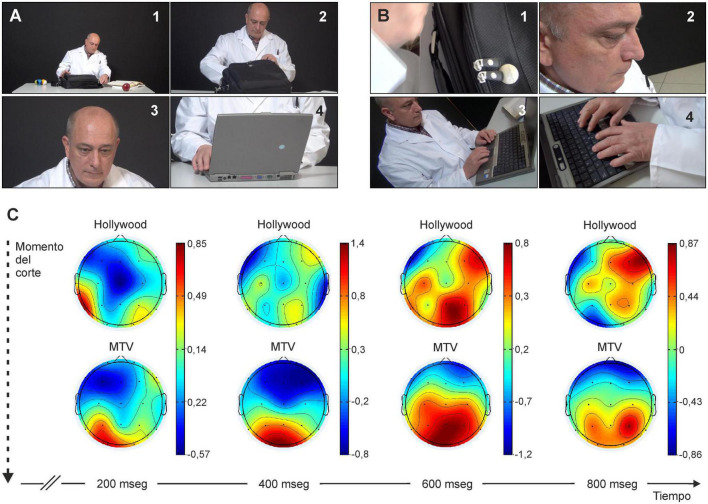
For a visual stimulus to be consciously perceived, the related neural activity must reach the prefrontal cortex. **(A,B)** Images from a short video filmed in Hollywood-style [each image lasts at least ≥ 5.9 s; in panel **(A)**] or in MTV-style [each image lasts ≈2.4 s; in panel **(B)**]. Experimental subjects indicate that they are unaware of perceiving many of the images shown in **(B)**. **(C)** Intensity of cortical activity from the start of a new image filmed according to Hollywood style panel **(A)** or MTV style **(B)**. Below in the figure is indicated the time elapsed since the start for the two experimental scenes. Note how visual activation does not reach the prefrontal zone in the case of MTV-style stimuli. Taken with permission and modified from Andreu-Sánchez et al. (2018).

Other things being equal, the duration of a stimulus significantly affects whether or not it reaches the conscious level. In a study carried out by our group, we have shown that the content of videos made following an ordered editing style, similar to that of classic Hollywood movies (with an average length of shots between cuts of 5.9 s) reaches the conscious level better than those editions made in the chaotic style typical of MTV-type video clips (takes with an average of 2.4 s). In our study (Andreu-Sánchez et al., 2018) we were able to verify that, although both types of visual stimulus reach and activate the visual cortex (located in the occipital portion of the brain), those performed in the Hollywood style reach the prefrontal cortex with greater intensity, a structure which is necessarily activated during the conscious perception of visual stimuli, while visual stimulus presented in the MTV style only reached the parietal area (Figure 4). In this regard, it is generally accepted that visual pathways in the cerebral cortex of primates are separated (although interconnected) in two different pathways: a dorsal stream, reaching parietal areas, mainly unconscious and specialized in the control of ongoing motor activities and a ventro-temporal pathway, further reaching prefrontal areas, mostly devoted to the construction of our conscious perception of the visual world (Milner and Goodale, 2008; Milner, 2012, 2017). In any case, there are numerous experimental studies in primates, including humans, on how subliminal perception, which does not reach the conscious level, can affect other perceptions, affective states, and decision-making (Thorpe et al., 1996; Kandel et al., 2013; Dehaene, 2014).

One of the first proofs of the localization in specific places of the brain of the conscious cognitive processes was the description of the areas of Broca (in 1861) and Wernicke (in 1876) in studies of certain language pathologies. Broca’s area is located in the lateral frontal lobe and it was described by Paul Pierre Broca after his postmortem studies of aphasic patients—i.e., this area was related to spoken language. In contrast, Wernicke’s area is located in the posterosuperior temporal lobe and it is related to the comprehension of spoken or written language, not to its oral expression, a function that relates to Broca’s area. Subsequent studies have shown that conscious cognitive processing (perceptions, thoughts, desires, affective states) requires the participation of different, strongly interconnected brain areas (Dehaene, 2014). In studies carried out in experimental animals (primates, cats, and rodents) it has been verified that the activity of individual neurons, located in the posterior parietal cortex or in the dentate nucleus of the cerebellum, are activated specifically when an intentional movement is made (of the hand or of the eyes) but not when the exact same movement is made unconsciously (Kandel et al., 2013). Finally, studies carried out on people in whom, for clinical reasons, the two cerebral hemispheres are separated (through the section of the corpus callosum that joins them) suggest that these patients present separate mental activities corresponding to the information present in each hemisphere, as if they happened to have two different minds (Cobb, 2020).

The conscious state always has a specific content, which is why it is accepted today that these contents change depending on the brain area active at that time. For example, if a person is shown an image of a face to one eye and a house to the other, the individual consciously alternates viewing one or the other, which he/she indicates by pressing a button. It is important to note that when you are looking at a face, the spindle-shaped facial area is active in your brain, while when you see the house, a different area is active: the parahippocampal spatial area. Thus, the conscious perception of a face or house is produced by activation of specific brain areas, while the visual cortex (non-conscious information) is activated in a similar way (Taylor and Villa, 2001; Hubel and Wiesel, 2004; Kandel et al., 2013). In any case, in the conscious state there is always an activation of the parietal and prefrontal cortices and a synchronized high-frequency electroencephalographic oscillation between all the cortical areas involved (Dehaene, 2014).

The conscious state evolves throughout the different vital stages as the brain structure and function change, and is modified by many phenomena that affect the brain, such as sleep, fever, and many different pathologies. Throughout the last quarter of the 20th century, Libet carried out a series of experiments to determine whether, in a voluntary act as simple as flexing a finger, the desire to do so or the activation of the corresponding area of the motor cortex occurred first (Libet et al., 1983; Libet, 1985). His conclusion was that the will to move the finger occurs two tenths of a second before the movement, while the preparatory potential registered at the cortical level precedes the movement by about five tenths of a second and, therefore, also precedes the conscious desire by about three. This result would be an experimental confirmation that neural activity precedes the conscious state and, of course, the movement to be executed. A more precise explanation of Libet’s results is the one proposed by Schurger et al. (2012) using a leaky stochastic accumulator to model spontaneous neural activities preceding self-initiated movements. For these authors, when the need to generate a movement is rather weak, the precise moment at which a decision to move is reached depends on spontaneous subthreshold fluctuations of neuronal activity and this process could take place at the same time as the conscious intention to move.

**FIGURE 5:**
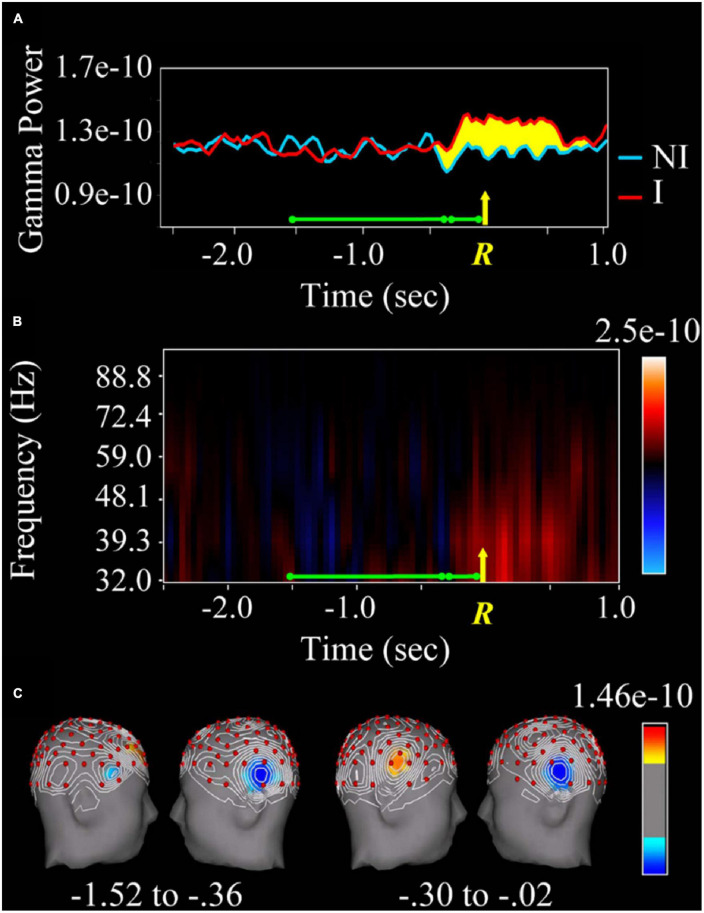
Changes in the gamma spectral band depending on whether or not the answer to a linguistic question is found. **(A)** Spectral analysis (in the gamma band) of the local field potential recorded in the right anterior temporal lobe during the resolution of the question (green line). The yellow R and the arrow indicate the moment of the manual response (pressure of a switch). The red line corresponds to the spectral power of the gamma band when the answer was guessed, while the blue one corresponds to the non-insight trials. The yellow-marked area is the difference between the two colored lines. Note that the activity starts 0.3 s before the switch is pressed. **(B)** Higher spectral power (indicated in red) of the gamma band (32–88.8 Hz) for insight versus non-insight plots. **(C)** The area of the right superior temporal lobe marked in red corresponds to the activation that occurs only when the task is correctly performed. Taken with permission from Jung-Beeman et al. (2004).

It is striking that while Libet’s experiments have been widely criticized from spheres that are not exactly neuroscientific, there have been few attempts to reproduce or refute them experimentally. In a recent series of experiments (Jung-Beeman et al., 2004; Truelove-Hill et al., 2018; Zhu et al., 2021), carried out with advanced recording techniques, the group led by J. Kounios has studied the brain activity related to the so-called “aha” moment (that is, the instant one feels accounts for the solution or explanation of a problem). The experimental subjects had to press a switch at the moment they found the solution to a word problem. The authors (see Jung-Beeman et al., 2004) found increased neural activity and high-frequency oscillation (39 Hz, gamma band) in the right-sided anterior superior temporal gyrus preceding the “aha” moment by one-third of a second. There is even earlier activity (approximately 1 s) at a lower frequency (9.8 Hz, alpha band) in the parieto-occipital area (Figure 5). Similar evidence of earlier activity patterns evoked in anticipation of an expected stimulus was reported in behaving rats and non-human primates (Villa et al., 1999; Villa, 2000).

The intrinsic neural mechanisms by which the brain generates consciousness are presently the matter of an intense debate (Melloni et al., 2021). As above indicated, visual processing of Hollywood style movies requires the activation of prefrontal networks, while most MTV style photograms remains mostly unconscious and, interestingly enough, these visual stimuli only reach parietal areas (Andreu-Sánchez et al., 2018). These results are in agreement with the global neuronal workspace theory (GNWT) (Mashour et al., 2020). The GNWT holds the simultaneous activation of prefrontal and parietal circuits, together with additional sensory cortical structures, to the generation of conscious states related to object or word recognitions. In contrast, the integrated information theory claims that, because its location and connectivity, the posterior cortex is ideally located to generate a maximum of integrated information able of generating a conscious state (Boly et al., 2017), a tenet not supported by our results. Obviously, more experiments carried out with more sophisticated recording devices will be necessary before we arrive to brain functional processes underlying the generation of conscious states.

## Emotional components of our internal world

It is more than common to forget the sentimental components of our internal world, as if decision-making or conscious processing of information from the sense organs or that stored in the form of emotional memories or knowledge acquired over a lifetime were not always colored and modulated by our emotional state. In the last third of the last century, neuroscientists almost unanimously accepted that the functions related to basic emotions (such as fear, love, depression, hate, or envy) were generated, processed, and stored in the so-called limbic system (Isaacson, 1982). The limbic system received this name because it was composed of a set of neural structures that formed a kind of circle or limbo around the diencephalon—the thalamus in particular. The limbic system is made up of the most primitive part of the cerebral cortex (paleo- and archi-cortices) including the hippocampus, the cingulate gyrus, the entorhinal cortex, and subcortical centers such as the amygdaloid nucleus and septum (Papez, 1937; Leventhal and Tomarken, 1986; Spyer, 1989). From the evolutionary point of view, this neural system is especially developed in birds and mammals. Activities as elementary as the control of aggressiveness or the explicit care of the young are important functions of this cerebral cortical structure (MacLean, 1952; Delgado García et al., 1998).

An important advance in the organization of the emotional world was the discovery of points or zones of positive or negative reinforcement in the nervous tissue. Since the middle of the last century, and from the studies of Olds, Milner, and many other researchers (see Olds, 1958), it has been discovered that the activation by means of electrical stimuli of areas such as the amygdala causes unpleasant emotional reactions (or punishment) in the animal, leading to the triggering of attack or flight responses (Delgado et al., 1954). In contrast, stimulation of nuclei such as the medial septum or the accumbens nucleus produces reactions that can be interpreted as pleasurable, or rewarding (Olds and Milner, 1954). This has been demonstrated experimentally (in mouse or rat) by the animal’s learning to carry out an activity such as pressing a lever repeatedly, in the first case to avoid receiving electrical stimuli in the amygdala or, conversely, pressing the same lever to receive electrical stimuli in the septum or in the accumbens nucleus (Jurado-Parras et al., 2012; Vega-Flores et al., 2014). Recent studies in primates, including the human species, have confirmed the role of these nuclei in defining the emotional components of behavioral and cognitive activities (Berridge and Kringelbach, 2008).

In later years and with the development of more-specific instrumentation for the study of the emotional behavior of mammals, specialized functions of the constituent structures of the limbic system and others associated with it have been determined. At this point, there is special interest in the birth of a social Neuroscience aimed to study how the brain of mammals makes possible the generation of behavioral functions such as cooperation, aggressiveness, empathy, or oral language (Kandel, 2012). Thus, it is now accepted that the social functions developed by primates are elaborated and regulated by neural circuits that link the hippocampus and the accumbens and amygdaloid nuclei with defined portions of the prefrontal and anterior cingulate cortex. In the case of the identification of specific individuals by their face and general appearance, the participation in these emotional control circuits of a series of specific neuronal centers located in the superior temporal sulcus has recently been discovered. While the amygdala deal with the emotional component of social interactions, the hippocampus performs other functions related to the acquisition and storage of different types of memory, and the accumbens nucleus determines the degree of satisfaction of appetitive, consummatory, and addictive behaviors (Spiga et al., 2011). Finally, the prefrontal cortex coordinates all these functions and organizes both the sequences of motor acts of the different behaviors and the related cognitive processes. In summary, today it is assumed that these neural circuits actively influence social decision-making, in both primates and rodents (Gangopadhyay et al., 2020).

## How the brain regulates behavioral, cognitive, and emotional functions

Behavioral and cognitive functions (unconscious and conscious), as well as their emotional components, are regulated from various neuronal groups located in brainstem centers. Due to their location, these centers project upward to different cortical and subcortical structures according to the specific functions in which they participate (Kandel, 2012).

The most general of all of them is the cholinergic (so-called because it releases the neurotransmitter acetylcholine), which mainly regulates the circadian rhythm of wakefulness and sleep, as well as all brain functions related to wakefulness, such as attention processes, and the acquisition of learning and its subsequent storage in the various types of memory. This system also (indirectly) participates in reward mechanisms because of its regulatory role with regard to the dopaminergic system. These cholinergic neurons are located primarily in the basal forebrain. The loosely used term basal forebrain refers to the approximate area at and near the inferior surface of the telencephalon, between the hypothalamus and the orbital cortex. The basal forebrain is located under the inferior surface of the rostral telencephalon, between the orbital cortex and the hypothalamus (Phillis, 2005).

Norepinephrine is another ascending system closely related to attention processes and alertness levels (Suárez-Pereira et al., 2022). Noradrenergic neurons are located in the so-called locus coeruleus, situated in the brainstem. These neurons are activated in stressful situations or in the presence of severe pain. The release of norepinephrine increases the level of vigilance, even producing the sensation of fear if it is released in considerable concentrations.

For its part, serotonin is a neuromodulator also present in invertebrates in which it participates in learning and memory processes. Serotonergic neurons are located in small nuclei throughout the brainstem and project to various limbic structures as well as other areas of the cerebral cortex. Serotonin’s role is related to the level of alertness and it is known that its deficit produces depressive states (Robbins, 2005).

A fourth ascending modulatory system is the dopaminergic system. This projecting neural system receives its name because it uses dopamine as a neurotransmitter. Dopaminergic neurons are located in the ventral tegmental area and in the substantia nigra. While the neurons of the ventral tegmental area project in particular to the prefrontal cortex, the hippocampus, and the accumbens and amygdaloid nuclei, and are concerned with the regulation of mechanisms of reward (pleasant sensations) or punishment (unpleasant sensations), the neurons of the substantia nigra project mainly to the basal ganglia, participating in the initiation and performance of motor activities designed in the premotor and motor cortices (Olds, 1958; Schultz, 1998; Cabessa and Villa, 2018).

Oxytocin and vasopressin are neurohormones present in invertebrates and vertebrates in which they fulfill very diverse functions related to the excretory and reproductive systems. However, recently, these neurohormones (particularly oxytocin) have been related to the processes of interpreting the intentions and desires of the fellow humans with whom we interact, as well as in situations of decision-making and cooperation between members of the same species. These neurohormones act by inhibiting the amygdaloid nuclei and activating specific areas of the prefrontal cortex, thus facilitating social interactions and sexual behaviors such as mating and caring for the young (Kosfeld et al., 2005; Donaldson and Young, 2008). Recently, the possible role of hypocretin-secreting neurons, located in the lateral hypothalamus, which are involved in alert mechanisms and motivated behaviors in complex and stressful situations, has also been described (Tyree et al., 2018).

Finally, endorphins are peptides that act on brain receptors on which morphine and other opiate derivatives also act. In general, endorphins are synthesized in the hypothalamus and act by inhibiting pain and generating a global sensation of satisfaction and pleasure. Endorphins are released in emotionally intense situations or after performing exercises and tasks that are accompanied by highly motivated states (Pert and Snyder, 1973; Corder et al., 2018).

As indicated in this section, while the different levels of consciousness are determined from centers of the brainstem, the contents (that is, what it is perceived) is processed by different regions of the cerebral cortex. At the same time, neither behaviors, nor cognitive processes, nor emotions and feelings are molecular or neural phenomena. All of these functions have to be considered, from a neuroscientific point of view and, as is the case with behavior, as emergent properties resulting from the activity of different nervous systems, including ours.

## Brain activity during social decision-making

From a neuroscientific perspective, the concept of free will (that is, the taking of different decisions in exactly the same environmental, social, and personal situations; see Mele, 2014) is difficult to approach experimentally due to the impossibility of accurately reproducing the same mental, behavioral, environmental, and social conditions at separate times. In any case, in his interesting and innovative book, Glimcher (2004) had already pointed out that human beings and animals are indeterminate physical systems—that is, capable of taking internal decisions independently of immediate stimuli, and of developing better- or worse-adapted responses to their physical and social environments.

**FIGURE 6:**
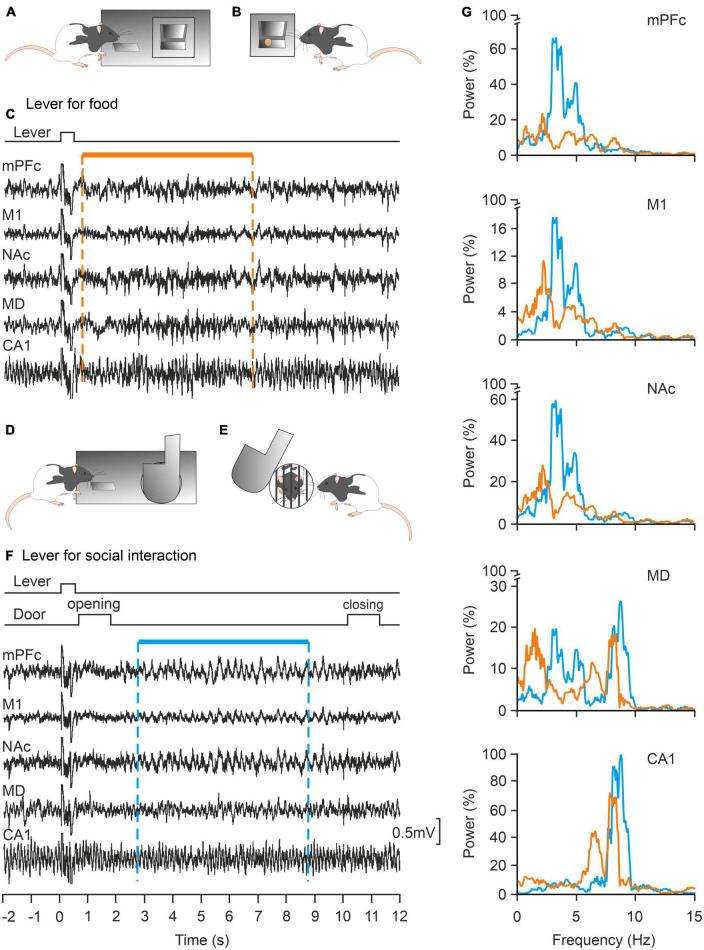
Local field potentials (LFPs) collected from cortical and subcortical structures of alert behaving rats are differentially involved in the same operant conditioning task depending on the expected reward: food or social interaction. **(A,B)** The experimental rat can press a lever panel **(A)** in order to collect a food pellet panel **(B)**. **(C)** Representative examples of LFPs recorded when pressing the lever for food are illustrated. **(D,E)** The rat can press a different lever panel **(D)** to open a door to interact with another littermate for a few seconds. **(E,F)** Representative examples of LFPs recorded when pressing the lever for social interactions. **(G)** Power spectra of LFPs (1–15 Hz) recorded during lever presses for food (orange traces) or social interactions (blue traces). Note the differences in power in the illustrated structures for the two experimental situations. mPFC, medial prefrontal cortex; M1, primary motor cortex; NAc, accumbens nucleus; MD, mediodorsal thalamic nucleus; CA1, dorsal hippocampal CA1 area. Taken and modified from Rocha-Almeida et al. (2018).

In contrast, the concept of decision-making is more approachable from an experimental point of view. As defined by Khani and Rainer (2016), decision-making “is an adaptive behavior that takes into account several internal and external input variables and leads to the choice of a course of action over other available and often competing alternatives.” Moreover, decision-making and risk-taking are affected by personality traits and cannot be accounted for by models based only on rational computations (Mesrobian et al., 2015). In our laboratory, we have developed an experimental model for the study of decision-making in rodents (rat and mouse). In one of the cases, the experimental animal is located in a room equipped with two levers. By pressing one of them the animal has access to a small piece of food, while by pressing the other the animal can have access to an adjoining compartment in which it can interact for a few seconds with a congener of the same sex. Taking advantage of this experimental approach (Rocha-Almeida et al., 2018) we have shown that the activity of the medial prefrontal cortex of the rat presents a completely different neural activity when pressing the lever to obtain food than when pressing the other lever to interact with another rat (Figure 6). A recent study carried out in behaving mice has shown that the medial amygdala also plays an important role in the reinforcement of this type of social interaction (Hu et al., 2021). These preliminary results suggest the participation of this structure in this decision-making situation. In a similar design, widely used in studies of the autistic syndrome, a mouse has the option of interacting with either an inanimate object or a conspecific of the same sex (Jabarin et al., 2022).

**FIGURE 7:**
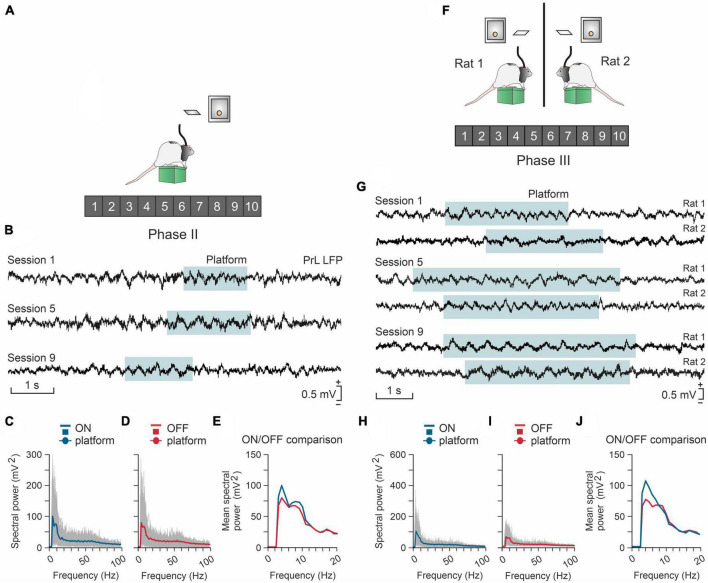
The prelimbic (PrL) cortex is differentially involved in the individual **(A–E)** or cooperative **(F–J)** acquisition of the same operant conditioning task. **(A)** A rat (#1) has to climb onto a platform and stay on it >0.5 s to activate a lever, and then to press it to obtain a reward (a food pellet). **(B)** Local field potentials (LFPs) recorded from the PrL cortex of the rat across three different sessions. The cyan-shaded areas indicate the time the rat was on the platform. **(C)** Spectral analysis for LFPs acquired from the rat while it was on the platform. Gray curves represent the spectrum of single trials, while the blue curve is the averaged spectrum. **(D)** Same as in panel **(C)** but with epochs acquired when the rat was off the platform. Gray curves represent the spectrum of single trials and the red curve is the averaged spectrum. Panel **(E)** a comparison of the averaged spectra for on- and off-platforms represented in panels **(C)** and **(D)**. **(F)** Two rats (#1 and #2) have to climb onto individual platforms and stay simultaneously on them > 0.5 in order to each activate a lever, and to press them to obtain a reward (a food pellet to each rat). **(G)** LFPs recorded in the PrL area of the two rats across three different sessions. The cyan-shaded area indicates the moments when the rats were simultaneously on their platforms during the cooperation task. **(H)** Spectral analysis for LFPs of 1 s epochs (NT = 300) acquired from three pairs of rats when they were simultaneously ON their platforms. Gray curves represent the spectra of single trials, while the blue curve is the averaged spectrum. **(I)** Spectral power for LFPs of 1 s epochs (NT = 312) acquired when the rats were OFF the platform during the simultaneous task sessions. Gray curves represent the spectra of single trials, while the red curve is the averaged spectrum. **(J)** A comparison of the averages for ON-platform epochs against the OFF-platform ones represented in panels **(H,I)**. Note that higher values of spectral power are observed around 3–6 Hz for the ON-platform condition. Taken and modified from Gruart et al. (2017) and Conde-Moro et al. (2019).

Above we have already pointed out the importance that the development of a social Neuroscience is taking on today (Welsh and Estes, 2018). In this sense, we have recently published a study in which laboratory rats given the possibility of obtaining small pellets of food if they voluntarily act jointly (Conde-Moro et al., 2019). To do this, two rats are placed in adjoining cages, separated by a grid so that they can see and smell each other, but not change cages. Each cage is fitted with a raised platform. The two rats have to get on its platform at the same time and to remain on it for 1 or 2 s to gain access to a lever, which when pressed releases a food pellet to each of the animals. This activity is certainly complex and requires precise and coordinated behaviors between the two experimental animals. Once again, in this model of social cooperation we have revealed the participation of the prefrontal cortex of the animals participating in the test (Figure 7). Prefrontal-accumbens (Jurado-Parras et al., 2012) and prefrontal-amygdala (Gangopadhyay et al., 2020) circuits have already been proposed as being involved in social decision-making situations.

## Some concluding remarks

1. Neurosciences have been expanding their focus from the study of the macroscopic and cellular structure of the brain, as well as its most-basic electrical properties, to the consideration of its sensory and motor functions and, lately, also its cognitive properties.
2. The brain allows the perception of various sensory modalities (vision, hearing, touch, etc.) and their integration into a perceptual whole, as well as the development of sensory-motor plans that make it possible to carry out extraordinarily elaborate and selective behaviors.
3. Behaviors are generated by internal and private motivations of each individual, based on their elective needs.
4. The brain also develops unconscious and conscious activities depending on its functional states (sleeping, awake, attentive, thoughtful, etc.).
5. The brain has various neuronal systems, mediated by specific neurotransmitters and neuromodulators that underlie the regulatory processes of its behavioral, cognitive, and emotional functions.
6. The concept of free will presents evident conceptual difficulties so that it cannot be approached from an experimental point of view. In this sense, the concept of decision-making seems more approachable.
7. The human brain and that of many other species makes it possible (by means of functional processes that are currently becoming known) to develop complex functions such as decision-making in ambiguous, compromised, or conflicting situations.

In conclusion, deciding is up to one person, since I and my brain are two sides of the same thing, or, in the form of an equation, brain = behavior + mental activity.

## Ethics statement

Written informed consent was obtained from the individual(s) for the publication of any identifiable images or data included in this article.

## Author contributions

Both authors prepared the illustrations and wrote the manuscript, contributed to the article, and approved the submitted version.
